# A replication study of GWAS findings in migraine identifies association in a Swedish case–control sample

**DOI:** 10.1186/1471-2350-15-38

**Published:** 2014-03-28

**Authors:** Caroline Ran, Lisette Graae, Patrik KE Magnusson, Nancy L Pedersen, Lars Olson, Andrea C Belin

**Affiliations:** 1 Department of Neuroscience, Karolinska Institutet, Retzius väg 8, Stockholm 171 77, Sweden; 2 Department of Medical Epidemiology and Biostatistics, Karolinska Institutet, Nobels väg 12A, Stockholm 171 77, Sweden

**Keywords:** Association study, Illumina, SNP

## Abstract

**Background:**

Migraine is a common neurovascular disorder with symptoms including headache of moderate to severe intensity and recurring attacks. There is no cure for migraine today and the pathology is poorly understood. Common forms of migraine have a complex genetic background and heritability has been estimated to be around 50%. Recent genome-wide association studies (GWAS) on European and American migraine cohorts have led to the identification of new genetic risk factors for migraine.

**Methods:**

We performed an association study in a Swedish population based cohort, investigating the frequency of eight single nucleotide polymorphisms (SNPs) recently identified as genetic risk factors for migraine in three GWAS, using available array data (Illumina Omni Express chip). The eight SNPs were rs2651899, rs3790455, rs10166942, rs7640543, rs9349379, rs1835740, rs6478241 and rs11172113. Because information on rs3790455, rs10166942 and rs7640543 was not directly available, we selected SNPs in high Linkage Disequilibrium (LD) with these three SNPs, and replaced them with rs2274316, rs1003540 and rs4075749, respectively.

**Results:**

We were able to replicate the association with rs2651899 and found a trend for association with rs1835740 in our Swedish cohort.

**Conclusions:**

This is the first reported genetic association study of a Swedish migraine case control material. We have thus replicated findings of susceptibility loci for migraine in an independent genetic material, thereby increasing knowledge about genetic risk factors for this common neurological disorder.

## Background

Migraine is a common neurovascular disorder with a strong genetic component. Symptoms include headache of moderate to severe intensity and recurring attacks. Diagnosis is made according to the criteria of the International Classification of Headache Disorders (ICHD-II) from the International Headache Society (IHS) [1]. There are two main subgroups of the disorder: migraine with aura (MA) and migraine without aura (MO), with the latter being the most common subtype. Aura is defined as a period with variable focal neurological symptoms, most commonly affecting vision and occasionally also the sensory system, that precede and sometimes accompany the headache phase. Many patients having migraine with aura sometimes also experience attacks without aura. In concordance with the global lifetime prevalence of migraine, which has been reported to be 14% [2], the lifetime prevalence of migraine in the Swedish population is 13,8% [3], which corresponds to more than one million people. Thus, migraine is a major neurological disease of considerable public health relevance. There is a female to male predominance, with a peak in middle aged women [2,4]. There also seems to be some regional differences in prevalence with more migraine patients in Europe and North America compared to the rest of the world [2]. Having relatives with migraine is a strong risk factor and heritability has been estimated to be as high as 50% [5].

It is not understood how migraine attacks are initiated. The symptoms of migraine headache are commonly hypothesized to be caused by activation of trigeminal nerve fibers and the C2 nerve fibers that coil around the large arteries of the brain and release of vasodilating chemicals such as calcitonin-gene-related peptide (CGRP) [6,7]. Activation of these nerves is assumed to cause pain and inflammation. Sensitization of the primary afferent nociceptive neurons innervating the vessels and the dura mater may increase their responsiveness at the site of inflammation [8].

An alternative hypothesis for migraine headaches is increased brain excitability. This is supported by genetic findings in familial hemiplegic migraine (FHM), a rare monogenic subtype of MA, where mutations are found in ion channels, potentially influencing cellular excitability [9,10]. This is in agreement with the finding that topiramate, a calcium channel blocker generally used to treat epilepsy, has been shown to be effective in migraine as well [11]. Genetic studies in the common forms of migraine have had limited success in the past decade. However, in three recent genome-wide association studies (GWAS) on large European migraine cohorts, a first set of genetic risk factors for migraine has been identified [12-14]. In the first study by Anttila et al., the single nucleotide polymorphism (SNP) rs1835740 was identified as associated with migraine in a two stage GWAS in seven North European migraine populations [12]. A strong association with migraine was found for the minor allele of rs1835740 on 8q22.1 (p = 5.38 × 10^−9^). This genetic risk factor was stronger for MA, than MO. The SNP rs1835740 is located in a 27 kb haplotype block between two genes involved in glutamate homeostasis, *MTDH* (metadherin, astrocyte elevated gene 1) and *PGCP* (plasma glutamate carboxypeptidase). Anttila et al. further performed an expression quantitative trait locus (eQTL) analysis which revealed that the rs1835740 risk allele was associated with higher *MTDH* expression. In addition, *MTDH* was also recently found to be associated with migraine in a genome-wide meta-analysis including six population-based European cohorts [15]. The second GWAS was performed on a cohort consisting of more than 20,000 American women of European descent. Results were replicated in two smaller independent samples of European migraine patients [13]. Three associations were found; rs2651899, rs10166942 and rs11172113, none of which were preferential for MA or MO. Rs2651899 is located in the gene *PRDM16* (PR domain containing 16), a zinc finger transcription factor whose potential role in migraine is unclear. Rs10166942 is located close to the transcription start site for *TRPM8* (Transient receptor potential cation channel, subfamily M, member 8). *TRPM8* is a sensor for cold mainly expressed in the peripheral nervous system, and is likely to be involved in neuropathic pain [16]. Rs11172113 maps to *LRP1* (Low density lipoprotein receptor-related protein 1), an endocytotic receptor protein involved in multiple cellular functions, such as modulating neuronal glutamate signaling [17] and lipid homeostasis [18], as well as being involved in the clearance of apoptotic cells [19] and amyloid precursor protein [20]. Findings of association with both rs10166942 and rs11172113 were recently replicated in a Scandinavian cohort consisting of more than 2,500 migraine patients [21]. The third GWAS was performed on German and Dutch migraine patients without aura, and the material partially overlapped with the Anttila study [14]. The study found associations with four new loci: *MEF2D* (myocyte enhancer factor 2D), *TGFBR2* (transforming growth factor β receptor 2), *PHACTR1* (phosphatase and actin regulator 1) and *ASTN2* (astrotactin 2), and also replicated the previously reported associations with *TRMP8* and *LRP1*. The SNPs with the strongest association at the respective locus were: rs3790455 (*MEF2D*), rs7640543 (*TGFBR2*), rs9349379 (*PHACTR1*), and rs6478241 (*ASTN2*). *MEF2D* is a transcription factor that promotes the survival of newly formed neurons in the brain [22]. *MEF2D* also influences the number of excitatory synapses in an activity dependent manner, and might thus influence neuronal excitability, which is an appealing role for a migraine candidate gene. *PHACTR1* regulates the activity of PP1 (protein phosphatase 1), which is known to influence synaptic activity and morphology [23,24]. *TGFBR2* is a serine-threonine kinase involved in proliferation, and *ASTN2* codes for a protein expected to influence neuronal migration [25,26].

Due to previous findings of mutations in ion channels causing different forms of FHM, genetic studies of migraine have up until recently mainly been limited to these areas of the genome. However, recent advancements in high-throughput genotyping technology have enabled more hypothesis free searches of the whole genome, revealing new susceptibility loci for migraine. Findings from these GWAS should be replicated in independent populations, since genetic risk- or protective factors can vary between different populations. Therefore the aim of the present study was to perform a replication study in a Swedish cohort on eight SNPs identified as genetic risk factors for migraine in large European and American GWAS.

## Material

The genetic material, consisting of 9,897 Swedish individuals, was collected by the Swedish Twin Registry. We declare that all experiments on human subjects, human material and human data have been performed in accordance with the Declaration of Helsinki. All procedures were carried out with the adequate understanding and written consent of the subjects. Formal approval to conduct the experiments described has been obtained from the human subjects ethical review board of Stockholm (reference number 2007/644-31). The occurrence of migraine was self-assessed through a questionnaire using the criteria for migraine of the ICHD-II [1]. Participants were asked to address the following questions to be answered “yes”, “no” “do not know”, or “don’t want to answer”: 1) recurrent headache not related to infection, fever or alcohol consumption (past or present); 2) headache attacks somewhere between 4 hours and 3-days if no medication against the pain is taken; 3) at least two out of four pain features (moderate or severe intensity, one-sided location, pounding/throbbing quality, and aggravation by routine physical activity; and 4) at least one out of two accompanying symptoms (nausea and/or vomiting, and increased sensitivity to light and sound) [5]. Information on migraine with aura could be retrieved from follow-up questions also answered by “never”, “sometimes”, “always”, “don’t know”, or “don’t want to answer”: Do you usually just before the headache begins have any of the following symptoms: visual disturbances, blurred vision, see flashes, flickering or zigzag lines, have difficulty reading or loss of letters. These symptoms must persist for at least 5 minutes and maximum 1 hour.

## Methods

The material includes both patients with and without aura, but for most subjects, information on aura is missing and the two groups were therefore not analyzed separately in our study. 910 individuals were classified as having migraine according to ICHD-II and 7,544 were not. The remaining 1,443 individuals, for whom this information was not available, were not included in this study. Genotyping was done on the Illumina Omni Express chip at the SNP&SEQ Technology Platform, Uppsala University. After an initial quality control performed by the platform core facility, controlling for missingness (max 0.03 genotypes missing per-SNP), low genotyping rate (max 0.03 genotypes missing per-individual), SNP frequency (minor allele frequency < 0.01), genetic sex, Hardy Weinberg equilibrium (p ≤ 1e^−07^) and heterozygosity, data from 644,556 single nucleotide polymorphisms (SNP) remained.

We performed cryptic as well as subject relatedness analyses, making sure there were no unknown relations in the material, which resulted in the removal of 137 subjects. In order to perform an association analysis we selected one twin from each pair, keeping the twin with migraine when diagnosis was discordant. From concordant pairs, one twin was randomly selected. A population stratification analysis based on MDS (minimum distance separation) calculations in four dimensions revealed one outlier, which was also removed (defined as deviating more than 6 standard deviations from the mean), leaving 749 cases (78% females) and 4,018 controls (47% females). After quality control we performed a one-degree of freedom allelic chi-square (χ^2^) test and calculated the genomic inflation factor λ to 1.005.

Three of the selected SNPs, rs3790455, rs10166942, and rs7640543, had not been genotyped in our material, why we instead included other genotyped SNPs, rs2274316, rs1003540, and rs4075749 respectively, in high linkage disequilibrium (r^2^ > 0.89) with the initially selected ones (Table 1). Thus genotyping data for rs2651899, rs2274316, rs1003540, rs4075749, rs9349379, rs1835740, rs6478241, and rs11172113 were extracted for all 749 cases and 4,018 healthy controls. Power analysis revealed that, with our sample size we should be able to detect effect sizes with odds ratios <0.753 and >1.294 with a minor allele frequency (MAF) of 0.22 in controls (power = 80% and α = 0.05). For the association test we performed a logistic regression analysis using gender as cofactor to eliminate any bias introduced by the predominance of female migraine patients in our material.

**Table 1:** **Eight SNPs analyzed for association with migraine in a Swedish population**-**based cohort**

**Chr**	**SNP of interest**	**Alleles**	**Replacement SNP**	**Gene**	**Alleles**	**Risk Allele**	**MAF**	**OR**
1	rs2651899	T > C		*PRMD16*		C	0.45	1.10
1	rs3790455		rs2274316	*MEF2D*	A > C	C	0.34	1.23
2	rs10166942		rs1003540	*TRMP8*	A > G	A	Na	Na
3	rs7640543		rs4075749	*TGFBR2*	T > C	C	Na	Na
6	rs9349379	A > G		*PHACTR1*		A	0.38	0.82
8	rs1835740	C > T		*MTDH*		T	0.22	1.0
9	rs6478241	G > A		*ASTN2*		A	0.38	1.22
12	rs11172113	T > C		*LRP1*		T	0.4	0.86

*Chr* = *chromosome*; *SNP of interest* = *SNPs identified in previous GWAS*, *see article text for references*; *Replacement SNP* = *SNPs of interest not genotyped on the Illumina chip were replaced with other genotyped SNPs in linkage disequilibrium with the SNP of interest* (*r*^*2*^ > *0.89*); *Gene* = *the SNPs are either located within or close to these genes*; *MAF* = *minor allele frequency from Freilinger et al*. [14]; *OR* = *odds ratio from Freilinger et al*. [14].

To evaluate the distribution of carrying more than one risk allele between cases and controls, an unweighted cumulative risk analysis for our eight selected SNPs was performed. Risk alleles were defined using odds ratios published in a previous study (Table 1) [14]. For two of the substituted SNPs (rs1003540 and rs4075749) we used HapMap Genome Browser (Phase 1, 2 and 3 merged genotypes and frequencies, build 37) at the International Haplotype Mapping Project web site (http://www.hapmap.org) to determine which proxy allele lies on the same haplotype as the published risk allele. Possible differences between control subjects and migraine patients were evaluated using a Chi-square test for trend.

Analyses were performed using PLINK v1.07 [27], GraphPad Prism version 5.03, (GraphPad Software, San Diego California USA), power and sample size software (PS version 3.0 http://biostat.mc.vanderbilt.edu/wiki/Main/PowerSampleSize) and R version 2.15.2, a free software programming language for statistical analyses [28].

## Results

We studied eight SNPs previously identified as genetic risk factors for migraine in European GWAS: rs2651899 (1p36.32), rs3790455 (1q22), rs10166942 (2q37.1), rs7640543 (3p24), rs9349379 (6p24), rs1835740 (8q22), rs6478241 (9p33) and rs11172113 (12q13.3) [12-14]. Because information on rs3790455, rs10166942 and rs7640543 was not available from the Illumina Omni Express chip data, we replaced them with genotyped markers in high linkage disequilibrium (r^2^ > 0.89). The replacement markers were rs2274316, rs1003540, and rs4075749 respectively, see Table 1. The disease-association test was performed with a logistic regression analysis using gender as a cofactor, with Bonferroni correction for multiple testing.

Allele frequencies and association results for all SNPs are shown in Table 2. We found that one of the SNPs (rs2651899), previously identified as a risk factor for migraine in Europe by Chasman et al. [13], was associated with migraine also in Sweden. The minor allele of rs2651899 was more common in cases than in controls, the association was significant before (p = 0.0019) and after Bonferroni correction (p_c_ = 0.0150). This association gave an odds ratio of 1.20 with a 95% confidence interval (CI) of 1.07-1.35 indicating that the minor allele confers an increased risk of migraine. We also found a trend for association with the minor allele of rs1835740 and migraine (p = 0.0075, p_c_ = 0.0600) with an odds ratio of 1.28, 95% CI of 1.07-1.54. The six remaining SNPs showed no statistically significant association with migraine in our material. All eight SNPs were in Hardy Weinberg equilibrium in control individuals (data not shown). A formal interaction test to investigate if the eight SNPs would have different effects in males and females showed no significant association (Table 3).

**Table 2:** Association results for the eight SNPs included in the analysis

**SNP ID**	**MAF cases**	**MAF controls**	**p-value**	**Corrected p**-**value**	**OR (95% CI)**
rs2651899	0.4519	0.4014	**0.0019**	**0.0150**	1.20 (1.07-1.35)
rs2274316	0.3598	0.3554	0.6143	1	0.97 (0.85-1.11)
rs1003540	0.1796	0.2069	0.0692	0.5538	0.81 (0.64-1.02)
rs4075749	0.3302	0.315	0.3961	1	1.06 (0.93-1.21)
rs9349379	0.4339	0.4389	0.4957	1	0.96 (0.85-1.08)
rs1835740	0.2109	0.1992	**0.0075**	0.0600	1.28 (1.07-1.54)
rs6478241	0.3758	0.361	0.6192	1	1.03 (0.91-1.17)
rs11172113	0.3865	0.4145	0.1569	1	0.92 (0.82-1.03)

*The disease*-*association test was performed using logistic regression analysis with gender as cofactor. SNP* = *Single Nucleotide Polymorphism; MAF* = *minor allele frequency*; *corrected p*-*value was calculated using the Bonferroni correction method*; *OR* = *odds ratio*; *95*% *CI* = *95*% *confidence interval. The significant p values are shown in bold (p < 0.05).*

**Table 3:** Allele frequencies in males and females separately

	**Females (n = 2471, nM = 587)**		**Males (n = 2296, nM = 162)**		
**SNP ID**	**MAF cases**	**MAF controls**	**MAF cases**	**MAF controls**	**Formal interaction test p-value**
rs2651899	0.4608	0.4095	0.4189	0.3942	0.4574
rs2274316	0.3646	0.3590	0.3426	0.3522	0.6326
rs1003540	0.1857	0.2123	0.1574	0.2022	0.4606
rs4075749	0.3311	0.3138	0.3272	0.3123	0.9317
rs9349379	0.4370	0.4429	0.4228	0.4353	0.8449
rs1835740	0.2129	0.1948	0.2037	0.2031	0.5064
rs6478241	0.3739	0.3718	0.3827	0.3515	0.3549
rs11172113	0.3978	0.4094	0.3457	0.4189	0.059

*n* = *number of individuals*; *nM* = *number of Migraineurs*; *SNP* = *Single Nucleotide Polymorphism*; *MAF* = *Minor allele frequency*; *Formal interaction test of the logistic regression performed with gender as covariate*.

**Figure 1:**
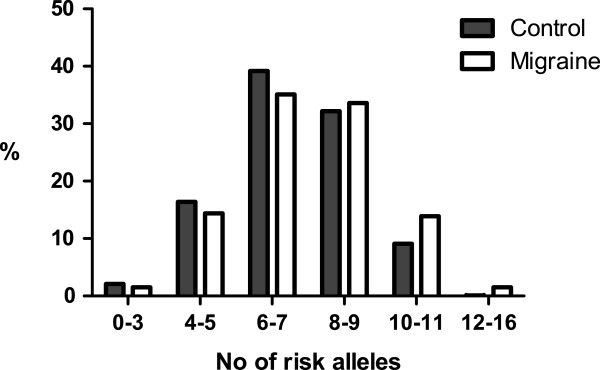
**Results from the unweighted cumulative risk analysis.** Differences in binned distributions of risk alleles for unweighted analysis were significant with a chi-square (Χ^2^) of 16.31 and a p-value < 0.0001. No = number.

In order to evaluate the distribution of risk alleles between migraine patients and controls, we performed an unweighted cumulative risk analysis for our eight selected SNPs. Most individuals had between five and nine risk alleles (82% of the controls and 80% of the patients). Subjects were divided into eight bins depending on their number of risk alleles (Table 4). The two bins with the lowest number of alleles (0–1 and 2–3) as well as the two bins with the highest number of alleles (12–13 and 14–16) were merged in the analysis in order to avoid bins containing zero individuals. Differences between control subjects and migraine patients were then evaluated using a (χ^2^) test for trend. Figure 1 shows that the control group had higher percentages of individuals carrying lower risk allele loads, whereas the migraine group generally had greater percentages of individuals carrying higher risk allele loads. The overall difference when comparing controls to migraine patients was significant with a (χ^2^) test for trend of 16.31 and a p-value < 0.0001.

**Table 4:** Distribution of risk alleles among controls and migraine patients

**Number of Alleles**	**Control % (n)**	**Migraine % (n)**
0-1	0 (2)	0
2-3	2.0 (82)	1.5 (11)
4-5	16.4 (660)	14.4 (108)
6-7	39.1 (1573)	35.1 (263)
8-9	32.2 (1293)	33.6 (252)
10-11	9.1 (366)	13.9 (104)
12-13	1.0 (42)	1.3 (10)
14-16	0	0.1 (1)

*n* = *number of individuals.*

## Discussion

We have performed an association study in a Swedish material on eight SNPs previously identified as genetic markers for migraine. This is the first reported genetic association study done in a Swedish migraine case control material. It is well known that women are more likely to develop migraine than men, and in our material 78% of the patients were women. We therefore performed the disease-association test by using a logistic regression analysis controlling for gender. We were able to replicate the finding of association with one of the markers: rs2651899. Moreover, we see a trend for association for rs1835740 with migraine. Our analyses indicate that the association of rs2651899 and the trend for association of rs1835740 was not confounded by the female predominance in this cohort. In addition we found that migraine patients overall were more likely to carry the previously identified risk alleles than the control subjects.

Rs2651899 was first reported by Chasman et al. [13] in a population-based cohort including migraine patients both with and without aura, which is in agreement with the background of the cohort used in our study. Rs2651899 has also been confirmed to be a risk factor for migraine in a meta-analysis including both clinical- and population-based cohorts [21] and in MO patients in a Chinese cohort [29]. The minor allele of this SNP was associated with an increased risk of migraine also in our Swedish cohort. The rs2651899 variant has in contrast to these results recently been reported to have a protective effect on migraine susceptibility in a North Indian population [30]. The allele frequencies that we report for this SNP (0.4014%), is consistent with that reported for Europeans by Hap Map. However, allele frequencies seem to vary slightly between populations and the minor allele, C, which is also the ancestral allele, is found to be more common in some populations of African and Asian descent. The divergence of results observed here between a Swedish and an Indian cohort, might simply reflect a different genetic background. Genetic risk factors of migraine may also vary between populations, which is supported by the differences in prevalence described previously [2].

Rs1835740 was first reported as a risk factor for migraine by Anttila et al. [12] in a GWAS on seven European migraine case control materials from specialized headache clinics. The cases were divided into subgroups based on a diagnosis of MA or MO, and the effect of rs1835740 was stronger in individuals with MA. The North Indian study also reported significance for the heterozygous rs1835740 genotype in migraine patients (MO and MA) and more strongly so in MO [30]. Depending on the cohort background, different severity forms of the disorder may be represented, and therefore the underlying combination of genetic risk factors could also differ. Moreover, it has been suggested that the different phenotypes of migraine (e.g. with and without aura) represent different subgroups of the disorder with different genetic risk alleles involved in the underlying disease mechanisms. Therefore, it is interesting that this SNP also show a trend for association in the Swedish material, which is a population-based cohort including migraine patients both with and without aura. Note that the OR of 1.28 supports the hypothesis of the minor allele of rs1835740 conferring an increased risk of migraine. This OR is of a magnitude usually seen for common genetic variations in common disorders, and the 95% CI is clearly separated from zero. Considering our power analysis based on an OR of >1.294, this association would likely become significant if investigated in a slightly larger population.

Before the recently reported GWAS on migraine the search for candidate genes mainly evolved around ion channels. Mutations in ion channels have been shown to lie behind different forms of FHM [10,31], and the same genes were expected to influence all forms of migraine. In the recently published GWAS however, new groups of candidate genes have been proposed. *PRDM16*, which harbors rs2651899, is a zink finger transcription factor involved in the differentiation of brown adipose tissue in addition to repression of *TGF*-*β* (Transforming growth factor beta) signaling [32,33]. How *PRDM16* might contribute to migraine pathology remains to be elucidated. Positive GWAS findings indicate genetic loci that are associated with disease, while the true risk variant might be another SNP in strong linkage disequilibrium with rs2651899. Another possibility could be that rs2651899 is linked with a copy number variant or another structural variation causing the disease. An interesting follow up on this marker would be to use next-generation high-throughput sequencing technology and sequence a large number of cases carrying this variant in order to study this region more in detail.

Rs1835740 is located in a haplotype block between two genes involved in glutamate homeostasis, *MTDH* and *PGCP*. Anttila et al. performed an eQTL analysis which revealed that the rs1835740 risk allele was associated with higher *MTDH* expression [12]. MTDH has mainly been studied regarding its involvement in carcinogenesis, but has also been shown to downregulate *SLC1A2* (Solute carrier family 1, member 2), a major glutamate transporter in the brain. Down regulation of this gene leads to increased glutamate levels in the synaptic cleft and *SLC1A2* knockout mice have been shown to suffer from lethal spontaneous epileptic seizures [34]. Thus, the *MTDH* variant can indirectly affect the transport of glutamate at the synapse, supporting a role for disturbed glutamate homeostasis in migraine.

There is a clear predominance of females amongst migraine patients, which could very well be reflected in gender-specific risk factor differences. There is one subgroup of female migraine patients for instance, where the migraine attacks are initiated by hormonal fluctuations [35]. As shown by the formal interaction analysis, however, the eight SNPs studied by us did not have significantly different effects in females compared to males. It would nevertheless be interesting to see larger migraine cohorts analyzed with correction for gender and/or gender specific strata to investigate if certain alleles are more important in female or male patients. It is also of importance to find out how these risk factors affect men and women differently and what pathological mechanisms are involved.

With the recent advances in genetic migraine research we might expect to find that different forms of migraine; for example with or without aura, associate stronger with certain risk factors. If so, varying symptoms coupled to specific genotypes may suggest that migraine should rather be viewed as a spectrum of disorders than as a single, well defined disease. Functional studies of genetic variants associated with the disease will be necessary to further investigate the molecular pathways of involved genes in order to develop better treatment strategies for migraine patients.

One limitation of our study is that the diagnosis of migraine in our population was self-assessed based on a questionnaire with the ICHD-II criteria for migraine and not evaluated by a clinician. While the fact that previously identified risk alleles were verified or implicated in the Swedish material suggests that our questionnaire-based identification of migraine sufferers had sufficient discriminative power, a sharper image of genetic risk may be obtained in the future if participants are directly interviewed by neurologists.

## Conclusions

Our association study of a Swedish population-based cohort including migraine patients with and without aura, focusing on eight previously identified risk SNPs in large European GWAS, showed rs2651899 to be significantly associated with migraine. This strengthens previous findings of this marker as a variant involved in migraine pathology.

## Competing interests

The authors declare that they have no competing interests.

## Authors’ contributions

CR, LG and ACB performed variant analysis and interpretation, and drafted the manuscript. All authors read and approved the manuscript.

## Pre-publication history

The pre-publication history for this paper can be accessed here:

http://www.biomedcentral.com/1471-2350/15/38/prepub
